# Patient safety in undergraduate medical education: Implementation of the topic in the anaesthesiology core curriculum at the University Medical Center Hamburg-Eppendorf 

**DOI:** 10.3205/zma001220

**Published:** 2019-03-15

**Authors:** Nicolas Hoffmann, Jens C. Kubitz, Alwin E. Goetz, Stefan K. Beckers

**Affiliations:** 1 University Medical Center Hamburg-Eppendorf, Center for Anaesthesiology and Intensive Care Medicine, Department of Anaesthesiology, Hamburg, Germany; 2 RWTH Aachen, Faculty of Medicine, University Hospital RWTH Aachen, Anaesthesiology Clinic, Aachen, Germany

**Keywords:** medical education, errors, patient safety, anaesthesiology

## Abstract

**Introduction: **The focus of public attention and health policy is increasingly being drawn to patient safety. According to studies, more than 30,000 patients die each year as a result of medical errors. To date, learning objectives such as patient safety have not played a role in the core curriculum for medical education in Germany. The National Competence-Based Catalogue of Learning Objectives for Undergraduate Medical Education contains a total of 13 learning objectives relating to this subject.

**Methods: **In a descriptive study, learning content was implemented within the “Operative Medicine” study block offered by the Faculty of Medicine at Universität Hamburg. The definition and occurrence of errors as well as strategies for dealing with and avoiding errors were set as the learning objectives for an interactive lecture, problem-based learning (PBL) case as well as the bedside teaching on anaesthesiology. Students were able to evaluate the lecture directly. During the simulator session on anaesthesia, the safety-relevant information that students requested from patients was compared with the questions asked by a control group in the previous trimester.

**Results: **The topic of patient safety could be integrated into the “Operative Medicine” curriculum through a number of minor changes to classes. The accounts of personal experiences and importance assigned to the subject were considered positive, while content perceived as redundant was criticised. In the simulator, the students appeared to request more comprehensive preoperative safety-relevant information than the control group.

**Conclusion: **The subject’s relevance, positive feedback and trend towards a change in behaviour in the simulator lead the authors to deem introduction of the topic of patient safety a success.

## Zusammenfassung

**Einleitung: **Patientensicherheit rückt immer stärker in den Fokus der gesellschaftlichen und gesundheitspolitischen Aufmerksamkeit. Untersuchungen zufolge erliegen jährlich mehr als 30.000 Patienten den Folgen von Behandlungsfehlern. Im Pflichtcurriculum des Medizinstudiums in Deutschland spielten Lernziele wie Patientensicherheit bisher keine Rolle. Der Nationale Kompetenzbasierte Lernzielkatalog Medizin von 2015 sieht nunmehr 13 eigene Lernziele vor.

**Methoden:** In einer deskriptiven Studie wurden Lerninhalte hierzu in den Studienabschnitt „Operative Medizin“ an der Medizinischen Fakultät der Universität Hamburg implementiert. Als Lernziele wurden Definition und Entstehung von Fehlern sowie Strategien zum Umgang und Vermeidung von Fehlern in einer interaktiven Vorlesung, einem Problemorientierten-Lernen-Fall sowie im Unterricht-am-Krankenbett Anästhesiologie vermittelt. Studierende konnten die Vorlesung direkt evaluieren. Im Unterricht im Anästhesie-Simulator wurden die Studierenden bei Abfrage von sicherheitsrelevanten Informationen mit einer Kontrollgruppe aus dem vorhergehenden Trimester verglichen.

**Ergebnisse: **Das Thema Patientensicherheit ließ sich durch einfache Lehrveranstaltungsänderungen in das Curriculum „Operative Medizin“ integrieren. Positiv wurden die Schilderung von persönlichen Erlebnissen sowie die Wichtigkeit des Themenfeldes rückgemeldet, dagegen redundant empfundene Inhalte kritisiert. Im Simulator schienen die Studierenden die präoperativen sicherheitsrelevanten Informationen vollständiger abzufragen als in der Vergleichsgruppe.

**Schlussfolgerung:** Die Relevanz des Themas, das positive Feedback und der Trend zur Verhaltensänderung im Simulator ermutigen die Autoren, eine Einführung der Thematik Patientensicherheit als gelungen zu betrachten.

## Introduction

In recent years, the subject of patient safety and dealing with treatment errors and near misses has increasingly attracted the attention of the general public, health policy and the media. This opportunity has thus been seized upon to implement the subject of patient safety in the core curriculum for anaesthesiology students at the University Medical Center Hamburg-Eppendorf. The corresponding author’s project within the Master of Medical Education programme (in Germany) was based on the “six-step approach” to “curriculum development for medical education” [1].

### Step 1: “Problem Identification and General Needs Assessment” [1] 

According to data from the American National Academy of Medicine from the year 2000, as many as 100,000 deaths per year in the USA can be attributed to medical errors [2]. Epidemiological data from Germany puts the annual number of deaths caused by incorrect treatment at approx. 30,000–80,000 [3]. Beside health impairments or death, patient harm also leads to increased healthcare costs along with legal disputes and therefore represents a considerable economic factor.

Although the saying “Everyone makes mistakes!” has become something of a universal statement among the general populace, in medicine the culture of infallibility often still prevails.

#### Step 2: “Targeted Needs Assessment” [1] 

The efforts to develop medical education in Germany further have been stepped up extensively since the start of the last decade. More and more learning content is being added, including communication skills, simulation teaching and modern imaging techniques.

As the doctors of tomorrow, the medical students of today are not immune to medical errors either. In addition to the health problems of the harmed patients (“first victims”), mistakes made by medical practitioners can also cause harm in the sense of emotional distress (“second victims”). Despite its immense significance for patients and physicians alike, the topic of patient safety has not yet found its way into the core curriculum for medical education. Just a few years ago, only a few reports were published on obligatory teaching content within a practical surgical block [4] or optional courses as an elective [5]. However, the World Health Organisation (WHO) published a patient safety curriculum guide in 2009 [6] and the German Association for Medical Education (GMA) released a catalogue of learning objectives in 2016 [7]. The National Competence-Based Catalogue of Learning Objectives for Undergraduate Medical Education (NKLM) [8] published in 2015 contains a total of 13 learning objectives relating to patient safety.

The term “patient safety” does not appear in Hamburg’s catalogue of learning objectives for the clinical curriculum in medicine (KliniCuM) at all and the word “error” appears only once. However, all practising physicians are confronted with patient safety every single day of their professional lives. The authors therefore saw a need to integrate the topics of patient safety and error management into the core curriculum despite the wealth of medical content that must be covered.

## Project description

As part of a descriptive study, the curriculum within thematic block 2 on “Operative Medicine” at the UKE was adjusted to include a new lecture, a new problem-based learning case (PBL case) [9] and an observation task during the bedside teaching in anaesthesiology, and then evaluated.

### Step 3: “Goals and Objectives” [1]

The underlying aim of a change to the medical education is to improve the outcome for patients [10]. This could be achieved through a reduction in medical errors by implementing error avoidance strategies and thus enhancing patient safety, for example. The specific aim of this project was to implement learning content on patient safety and error management in the core curriculum for medical education so that all students are required to reflect on the topic at least to a minimum extent during their studies. Goals were developed for the classes (see table 1 (Tab. 1)): topics from in-house training on patient safety as well as procedural instructions and presentations by the UKE’s Office for Quality Management were compiled for content selection, prioritised using a matrix analysis and allocated to classes (cf. table 1 (Tab. 1)).

#### Step 4: “Educational Strategies” [1]

Since no additional teaching time was available for new learning content in thematic block 2 in 2013, existing teaching time was used. A 60-minute lecture introducing the subject of airways was dispensed with. A new case was then developed for the longitudinal PBL tutorial during the trimester. Finally, three observation tasks on the subject of patient safety were added to the checklist of practical activities (“mini logbook”) used during the bedside teaching in anaesthesiology.

##### Interactive lecture: “To err is human”

This lecture in the first week of the trimester was held before the practical clinical components. Seven specific learning objectives were formulated and assigned to one of the taxonomy levels according to Bloom [11] (cf. table 2 (Tab. 2)).

This interactive lecture was developed according to the sandwich principle [12], whereby phases of collective and frontal learning alternate with processing phases. The lecture’s structure and content is detailed in attachment 1 . The reflective tasks during the processing phases aimed to increase learning success [13], for example by making students aware of their own fallibility by considering their own mistakes (“Which mistakes have I already made?”) and thus rendering them “receptive” to the topic (“law of readiness”) [https://en.wikipedia.org/wiki/Principles_of_learning cited 2018 Jun 11].

The lecture concluded by looking at the PBL case and a special observation task during bedside teaching. In addition to the processing phases during the interactive lecture, students should delve deeper into the teaching content through repeated confrontation (in the PBL tutorial and bedside teaching) (cf. attachment 1 ).

##### PBL case: “surgical mix-up”

In the longitudinal PBL tutorial within thematic block 2 on “Operative Medicine”, the previous case was concluded and a new, supervised PBL case discussed during a total of 10 three-hour sessions, each attended by 15 students and a medical tutor. An anaesthesiology case was replaced with a new, open PBL case involving a surgical mix-up for this purpose. The PBL case “Cut!...” (cf. attachment 2 ) describes the preoperative preparation of a patient prior to orthopaedic surgery on a lower extremity through to the first incision being made into the wrong leg after a side mix-up. Five specific learning objectives relating to term definition, factors conducive to errors, systematic error analysis and error avoidance as well as reflection on how to deal with errors were targeted. The learning objectives intentionally covered many of the same learning objectives as the interactive lecture to help enhance learning success through repetition (“law of repetition”) [https://en.wikipedia.org/wiki/Principles_of_learning cited 2018 Jun 11].

##### Observation task with a checklist during bedside teaching

For the bedside teaching, students already received a checklist of practical training content to complete in the past that provided structure. It took the form of a “mini logbook” that they had to keep for themselves (cf. attachment 3 ). The checklist was supplemented with three practical tasks to enhance the focus on content relating to patient safety:

- perform preoperative checks (incl. patient identification, nil-by-mouth, allergies, intubation hindrances, procedure identification, patient clarification);
- receive a demonstration of medication labelling;
- participate in a “team timeout”.

#### Step 5: “Implementation” [1]

At the UKE, the clinical curriculum in medicine (KliniCuM) was taught in trimesters in 2013. The clinical component for students in years 3–5 was divided into a total of nine elective “thematic blocks” (trimesters): six thematic blocks, one elective (subject) block and two free blocks. Thematic block 2 on “Operative Medicine” was selected as the “setting” into which the three classes should be integrated. The curriculum change was implemented during the second trimester (April–July) in 2013. All students of thematic block 2 could participate in the interactive lecture during the first week of the trimester. Students completed the PBL tutorial as well as the bedside teaching in anaesthesiology during the “Anaesthesia Week” held at different times during weeks 2–11 of the trimester according to a rotation plan.

#### Step 6: “Evaluation and Feedback” [1]

Feedback was requested from the students who attended the lecture “To err is human” immediately after the session. Using the “one-minute paper” method, they were asked to make a note of a keyword on an index card of what they found good about the class or where they saw scope for improvement. The consistency of the feedback collected in this way was reviewed and the responses grouped by topic wherever possible.

The students of thematic block 2 on “Operative Medicine” were moreover observed during the simulator session on anaesthesiology held at the end of “Anaesthesia Week” in order to gain an impression of the possible effect of curriculum development (intervention). The safety behaviour was observed of both the students in a control group from the trimester preceding the intervention (January–March 2013) and the intervention group itself. The class tutors documented the relevant safety questions that students asked (patient’s name and date of birth, nil-by-mouth, allergies, dental status, premedication, and planned procedure) before administration of the anaesthesia in the patient simulator (cf. attachment 4 ). The quantitative evaluation recorded the frequency that questions were asked. All students gave their verbal consent to the observation of their specific “patient safety performance” and to its documentation to analyse this as part of the curriculum evaluation.

In addition to the targeted evaluation, individual statements on the new class on patient safety were also provided as part of the usual evaluation of the thematic block on “Operative Medicine” at the end of the trimester. 

## Results

The intervention with new classes, the interactive lecture “To err is human” and the “Cut!...” PBL case on surgical mix-ups were implemented as planned during the second trimester from April to July 2013. The checklist for bedside teaching in anaesthesiology (“mini logbook”) with the three observation tasks was made available to all 122 students completing the trimester.

### Feedback from the interactive lecture

The lecture held on 15 April 2013 was attended by approximately 75 students. According to the lecturer’s subjective perception, the students were extremely attentive (quiet atmosphere), particularly at the start of the lecture during discussion of the three cases of treatment errors. Around 70% of the students participated in the interactive phases. 30 students used the “one-minute paper” feedback method. Based on repeated mentions, positive aspects included the lecturer’s open nature (33%), the case studies (30%), important topic (23%) and self-reflection (20%). However, 30% of students found the class to be too long and 17% thought it too theoretical.

#### Comments on the thematic block evaluation of the PBL case

Five comments were made on the “Cut!...” PBL case during the usual online trimester evaluation in July 2013. The content of the open design PBL case with just one single one-page case vignette was criticised as “too insubstantial”. The suggestion was made to design the PBL case as a guided case study comprising several pages and to combine it with another PBL case as necessary.

#### Simulator outcomes

**Figure 1:**
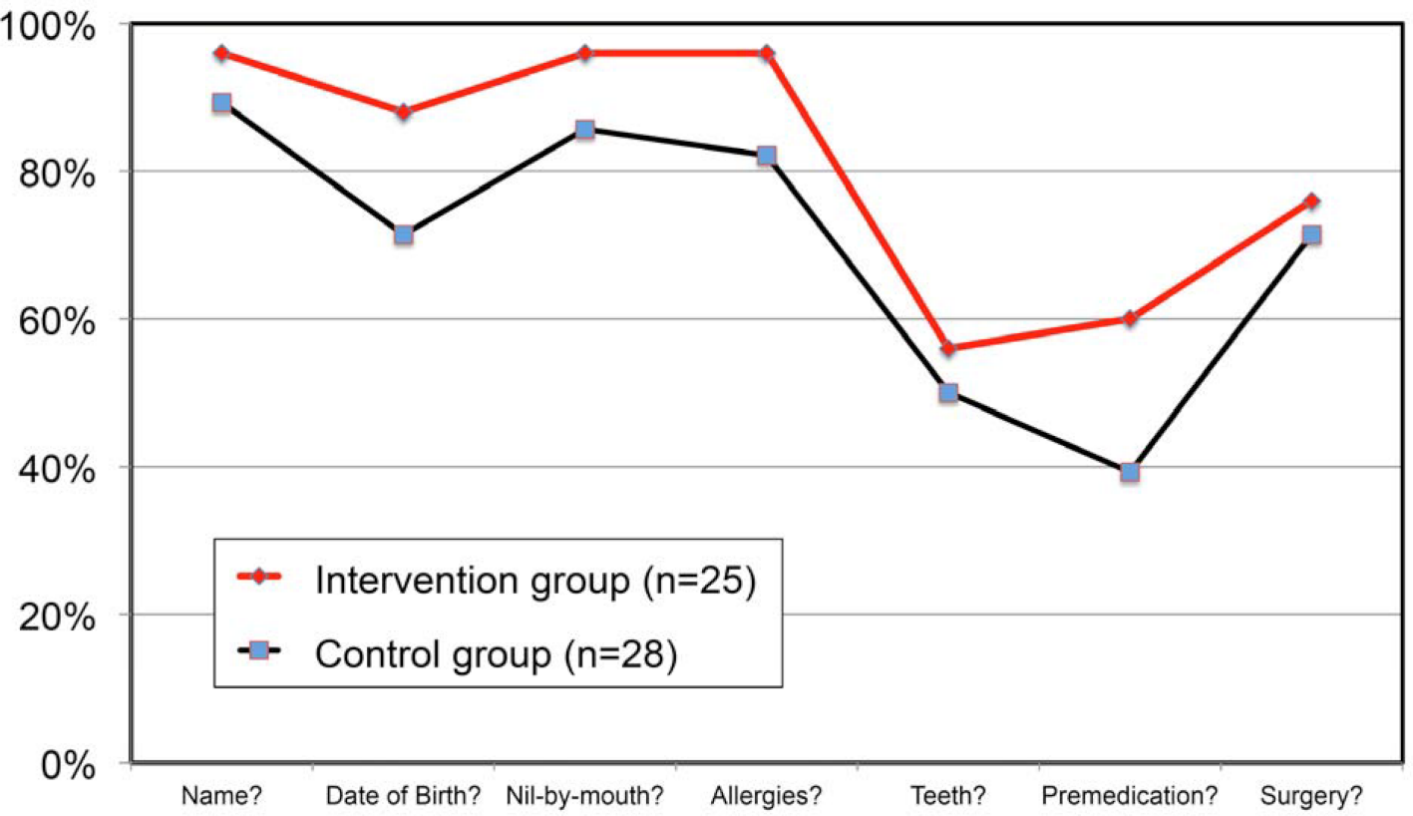
Descriptive presentation of the percentage of safety-relevant information requested before administering the anaesthesia during the patient simulator session

In the control group from the trimester preceding the intervention (January–March 2018), a total of 28 students were observed while they asked the safety questions during the preoperative check. In the intervention group, 25 students were observed (cf. figure 1 (Fig. 1)).

The percentage of safety information actually requested of patients was higher among the students in the intervention group for all seven items. In light of the insufficiently standardised study conditions, proof of statistical significance was deliberately forgone.

## Discussion

### Repeat of Step 1: “Problem Identification and General Needs Assessment” [1]

On the one hand, social and medical attention is increasingly being focused on the topic of patient safety. For treatment errors cause a significant number of deaths every year [2], [3]. On the other hand, the core curriculum for medical education, which is already packed with learning content, offers very little scope for the consideration of new aspects. The NKLM lists a total of almost 2,000 learning objectives [8], of which just 13 relate to or are associated with the topic of patient safety. Over a time-limited study period of six years [https://www.gesetze-im-internet.de/_appro_2002/BJNR240500002.html], experience has shown that the clinical disciplines tend to see a need for additional teaching time rather than willingly hand over teaching resources to other departments.

The project aimed to efficiently integrate the topic of patient safety into teaching at the Clinic for Anaesthesiology by exchanging a lecture, adding a PBL case study and complementing bedside teaching with an observation task.

All students taking thematic block 2 on “Operative Medicine” as part of the clinical curriculum in medicine (KliniCuM) in Hamburg could be familiarised with the topic of patient safety without having to add further teaching hours to the curriculum. To date, this has only been achieved during a three-hour compulsory module within the practical surgical block at the University of Greifswald [4]. Another published class described an in-depth compulsory elective module comprising 12 seminars [5], however this was only a pilot programme.

In terms of the structure, the minor changes could easily be implemented. The feedback from students during the lecture was positive and highlighted the need as well as the challenge of addressing this topic. Within the feedback provided during the lecture, positive emphasis was placed on the fact that this teaching content, which students considered important, is covered in class at all. The wish was expressed for more such classes. Several participants would like even more content on the procedure in the wake of a treatment error also involving harm to the patient, for example. In general, the feedback on the course (“one-minute papers”) was useful to the lecturer, as this course was offered for the first time and the feedback was very helpful for its further improvement.

The observation made during the anaesthesiology simulator session that students seem to be asking more detailed questions about important safety aspects before administration of the anaesthesia gave the positive impression that even the short amount of time spent discussing patient safety and error management could have led to increased awareness among the students of these issues.

The following aspects constitute limitations of this project: with the slight changes to the classes, nothing more than “minor goals” can be expected. The long-term learning effect of theoretical learning objectives and also of an interactive lecture are also expected to be as minimal. Even if efforts were made to consolidate the theoretical learning objectives by means of reflective tasks and the conscious repetition of content during the PBL case and bedside teaching, students’ “contact time” with the topic is simply too brief to be able to impart comprehensive concrete content.

An “open case” was developed for the PBL tutorial. The voluntary comments on the PBL case in the trimester evaluation revealed that students were unfamiliar with the concept of an “open” PBL case. Since “guided” PBL cases tend to be the rule in the clinical curriculum in medicine (KliniCuM), they were not acquainted with the approach to this case and this led to negative feedback. During the PBL tutorial, this case was linked to another “guided” anaesthesiology case study. The subjective impression was that students were better able to cope with this.

The three observation tasks listed in the checklist for bedside teaching in anaesthesiology are justified in terms of their content. However, experience has shown that checklists and logbooks are rarely used in clinical teaching [14]. A long-term learning effect therefore cannot be guaranteed.

The development of a catalogue of learning objectives on patient safety by the GMA committee for patient safety and error management on the national level [7] and by the WHO on the international level [6] reinforces the need to integrate the topic into the core curriculum. The entire content – for example, of a catalogue of learning objectives on patient safety for medical education by the GMA committee [7] – cannot be covered with the simple curriculum changes described. For it comprises three chapters containing a total of 38 learning objectives. However, 12 learning objectives from across all three chapters could be covered in this intervention (cf. table 3 (Tab. 3)).

The patient safety curriculum guide published by the WHO in 2009 provides comprehensive information on principles, objectives, structure and strategies to implement, review and evaluate a patient safety curriculum and details 11 topics [6]. Teaching time of 60–90 minutes is foreseen for each of these. Although seven of the 11 points categorised by the WHO (topics 1, 2, 5–7, 10 and 11) are explicitly addressed and covered in the project presented, the full scope of the WHO learning objectives cannot be conveyed in a 60-minute lecture, PBL case and observation task during bedside teaching. In a review published in 2015 on the effectiveness of patient safety curricula, a rather heterogeneous picture is painted of the course duration, structure and content of the 26 curricula innovations analysed [15]. In one third of these curricula, the course duration was approximately two to six hours – this was the amount of time assigned for the curriculum changes presented. That being said, one of the patient safety curricula analysed lasted an entire week, was based on the WHO learning objectives and covered all 11 topics [16].

The authors welcome the development of learning objective catalogues. Comprehensive implementation of these objectives is a worthwhile task, whereby this presents a huge challenge in view of the wealth of content needing to be covered during the core curricula. Implementation of this topic should be pursued in the future nonetheless. It is above all conceivable that partial aspects of this topic could be covered within other medical disciplines as part of a longitudinal curriculum on patient safety.

## Conclusion

The topic of patient safety is growing in importance. By publishing catalogues of learning objectives, various international and national institutions call for integration of the topic of patient safety into the core curriculum for medical education.

New content necessary for modern medicine, such as patient safety and error management, can be incorporated in with just a few changes to the curriculum and without adding new classes.

Even if a review of the learning objectives is challenging, targeted confrontation of the topic seems to be well received by students and regarded as important by them.

One limitation of this project is the lack of a statistical analysis as to whether the curriculum change represents significant added value for medical education (in the sense of a “justification study”). Due to the short teaching time, only a limited amount of the content listed in the catalogue of learning objectives on patient safety is covered.

The authors consider the project on implementing patient safety and error management in medical education as a first step towards introducing this important topic to be a success. As a consequence, the interactive lecture and PBL case study have been added to Hamburg’s new integrative model curriculum for medicine (iMED).

## Competing interests

The authors declare that they have no competing interests. 

## Supplementary Material

Plan for the interactive lecture according to the sandwich principle [12]

“Cut!...” PBL case on surgical mix-ups

Checklist for bedside teaching in anaesthesiology

Observation form for querying safety-relevant information prior to administration of the anaesthesia – “simulator observation”

## Figures and Tables

**Table 1:**
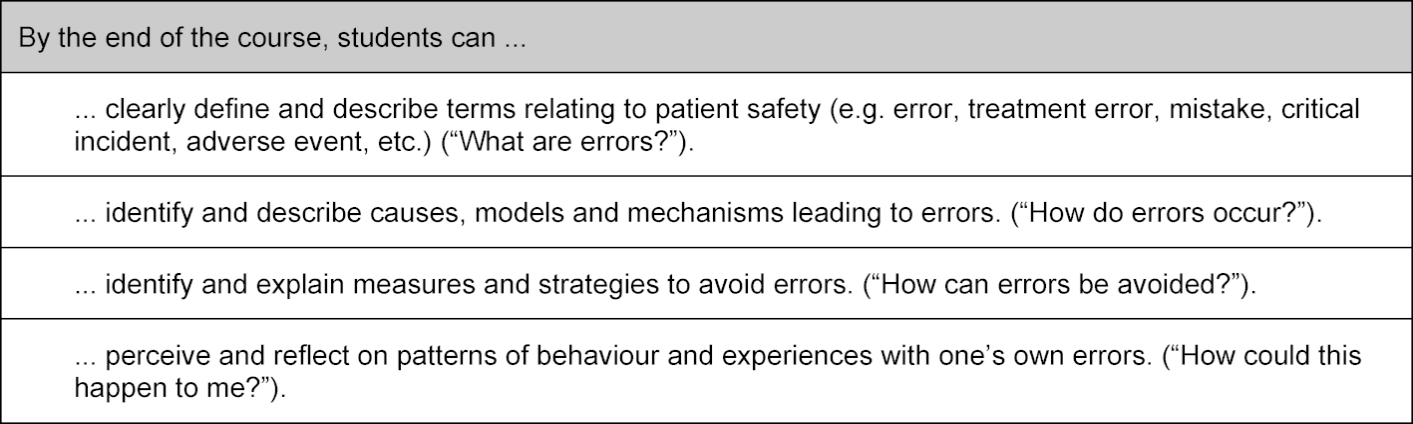
Goals for integration of the topics of patient safety and dealing with errors and near misses into the core clinical curriculum in medicine (KliniCuM) in Hamburg.

**Table 2:**
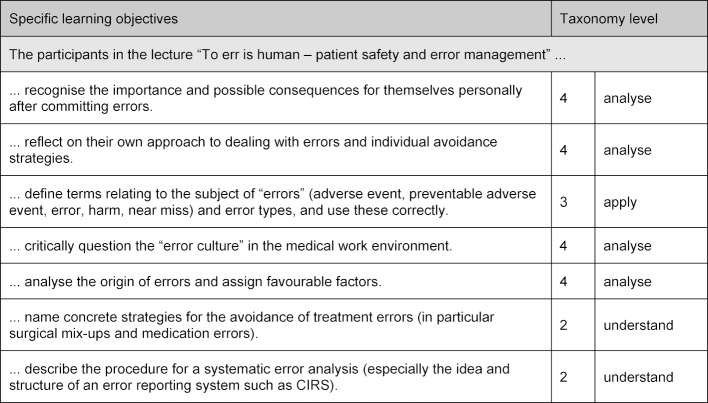
Each of the specific learning objectives has been linked to the related level and verb from the Bloom taxonomy [11].

**Table 3:**
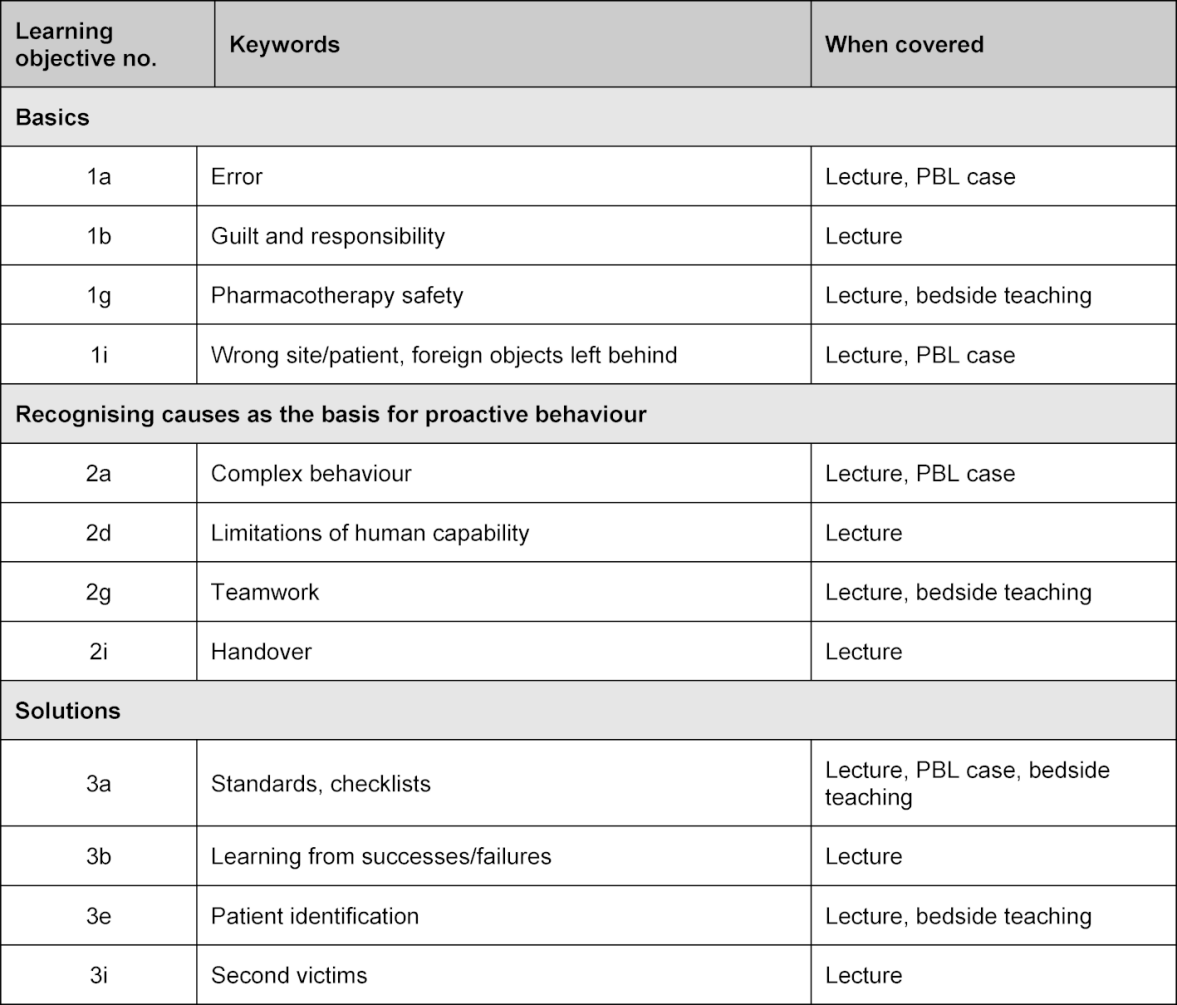
Learning objectives from the catalogue of learning objectives on patient safety for medical education by the GMA committee for patient safety and error management covered in class.
