# Supplementary material for: Patient safety in undergraduate medical education: Implementation of the topic in the anaesthesiology core curriculum at the University Medical Center Hamburg-Eppendorf
Source: GMS J Med Educ. 2019 Mar 15;36(2):Doc12. doi: 10.3205/zma001220 (PMC6446467; doi:10.3205/zma001220)
Supplement: Plan for the interactive lecture according to the sandwich principle [12] [file JME-36-2-12-s-001.pdf]

Phase	Content	Time
**A**<br>Introduction	Eye-catcher	
Activation; collective phase	Case presentations:<br>Case 1: a treatment error reported widely in the media<br>Case 2: a treatment error by a medical intern reported widely in the media<br>Case study 3: a personal case of a treatment error;<br>additional epidemiological information, social perception and consequences<br>-> should illustrate the topic's significance	10 mins
**B**<br>Learning break	Processing:<br>Reflective question for all participants:<br>"Make a note on a piece of paper of 1–3 errors that you personally have committed (e.g. in medicine, traffic, etc.)!"	2 mins
**C**	Information that these notes will be returned to later.<br>Transitional question: how are errors defined and how do they arise?	
Collective phase	Agenda + input:<br>First content block with definition of the terms error, error types and error culture. Sources of errors, favourable factors, models of error sources and risk areas.	15 mins
**B**<br>Learning break/partner work	Processing/consolidation:<br>Task for all participants:<br>"Discuss the personal events you have just made a note of with your neighbour:<br>- How would you describe these according to the definitions you have learned?<br>- At what point did the error occur (according to the model presented)?<br>- Categorise factors that led to errors as 'latent, conducive or active'?"	4 mins
**C**	Transitional question: Which strategies can be used to avoid mistakes?	
Collective phase	Input:<br>Second content block with the topics of systematic error analysis, measures to enhance patient safety through strategies to avoid treatment errors (especially surgical mixups and medication errors), presentation of projects to enhance patient safety (particularly error reporting systems such as CIRS).	15 mins
**B**<br>Learning break/partner work	Processing/consolidation:<br>Task for all participants:<br>"Continue to discuss your personal experiences with your neighbour:<br>- Which measures might have prevented the errors from occurring?"	4 mins
**C**	Voluntary report on participants' personal experiences	2 mins
**D**<br>Conclusion	Summary<br>Outlook for the coming trimester:<br>1. PBL case on patient safety	5 mins

	2. Formulation of observation tasks for the coming trimester:<br>- Observation task during the bedside teaching in anesthesiology in the operating theatre: "Which of the strategies presented were experienced?"<br>- Debriefing/report about it before/after the simulator seminar on anesthesia	
Evaluation	"One-minute paper" evaluation: students should make a note of a positive aspect on a white card and a point in need of improvement on a blue card as anonymous feedback.	3 mins
	Total	60 mins
