# Supplementary material for: Patient safety in undergraduate medical education: Implementation of the topic in the anaesthesiology core curriculum at the University Medical Center Hamburg-Eppendorf
Source: GMS J Med Educ. 2019 Mar 15;36(2):Doc12. doi: 10.3205/zma001220 (PMC6446467; doi:10.3205/zma001220)
Supplement: “Cut!...” PBL case on surgical mix-ups [file JME-36-2-12-s-002.pdf]

After much deliberation, 62-year-old Christian Schmidt has decided to undergo surgery. “I’ve suffered from pain in my left hip for more than five years now. The doctors say it’s arthrosis.” The day before the operation, the lead surgeon – Dr. Müller – comes to speak with the patient about implantation of a hip TEP. Mr Schmidt finds him “very pleasant and competent” and feels reassured about the upcoming operation. “He did forget his big felt-tip pen on my nightstand, though. But ... I suppose that can happen from time to time.”

On the morning of the operation, the surgical nurse greets Mr. Schmidt warmly and begins preparing him for surgery. Yes, he had slept well. The anaesthetist tells Mr. Schmidt he can “start thinking of a beautiful dream now”. The general anaesthesia is administered without any complications. Mr Schmidt can soon be wheeled into the operating theatre and the surgeon is informed that everything is ready for him.

After a short delay, another lead surgeon – Dr. Schulze – enters the operating theatre after surgical hand disinfection. “Morning all! Dr. Müller is off sick today, so I’m taking over for him.” Mr. Schmidt’s hip is swabbed with disinfectant and carefully covered. Dr. Schulze takes a scalpel in his hand, calls out “Cut!” in a loud voice and begins making an incision in the right hip …
