# Supplementary material for: Patient safety in undergraduate medical education: Implementation of the topic in the anaesthesiology core curriculum at the University Medical Center Hamburg-Eppendorf
Source: GMS J Med Educ. 2019 Mar 15;36(2):Doc12. doi: 10.3205/zma001220 (PMC6446467; doi:10.3205/zma001220)
Supplement: Checklist for bedside teaching in anaesthesiology [file JME-36-2-12-s-003.pdf]

Dear students,

The following checklist is intended as a guide for learning practical and theoretical skills during the bedside teaching in anaesthesiology.

Checklist for learning skills during the bedside teaching in anaesthesiology:

Name: ___________________________ ☑

- Basic monitoring set up ☐
- Preoperative checks performed (incl. patient identification, nil-by-mouth, allergies, intubation hindrances, procedure identification, patient clarification) ☐
- Intravenous cannula inserted (on anaesthetised patient) ☐
- Mask respiration performed ☐
- Demonstration received of the intubation procedure ☐
- Clinical examination performed (in particular, auscultation of heart and lungs, determination of pupil status) ☐
- Infusion prepared and demonstration received of medication labelling ☐
- Demonstration received of a transfusion as per the transfusion guidelines ☐
- Bedside test performed ☐
- Demonstration received of administration of a general anaesthesia ("9 steps") ☐
- Demonstration received of the basics of artificial respiration ☐
- Demonstration received of the basics of anaesthesia ☐
- Demonstration received of extended monitoring (arteries, CVC; indication and measured values). ☐
- Participated in "team timeout" ☐
