# Supplementary material for: Patient safety in undergraduate medical education: Implementation of the topic in the anaesthesiology core curriculum at the University Medical Center Hamburg-Eppendorf
Source: GMS J Med Educ. 2019 Mar 15;36(2):Doc12. doi: 10.3205/zma001220 (PMC6446467; doi:10.3205/zma001220)
Supplement: Observation form for querying safety-relevant information prior to administration of the anaesthesia – “simulator observation” [file JME-36-2-12-s-004.pdf]

Universitätsklinikum Hamburg-Eppendorf

Medical Clinic and Polyclinic for Anaesthesiology
Dr. med. Nicolas Hoffmann

January 2013

Observation form

Investigation of "patient safety in medical education"

Students in the anaesthesia simulator

Observation date: ☐

Scenario: ☐

Important note:
Please do not communicate the topic/questions to students (-> bias)!

Question	Asked?
Last name, first name?	☐
Date of birth?	☐
Nil-by-mouth?	☐
Allergies?	☐
Teeth/look inside mouth?	☐
Premedication received?	☐
Procedure, surgery site/side?	☐

In case of questions, please do not hesitate to contact Nicolas Hoffmann on (9)-50318!

Completed forms can be submitted to Nicolas Hoffmann's pigeonhole (copy room).

Thank you for your cooperation!

© UKE 2009
